# Expression of B7-H3, a Potential Factor of Tumor Immune Evasion in Combination with the Number of Regulatory T Cells, Affects Against Recurrence-Free Survival in Breast Cancer Patients

**DOI:** 10.1245/s10434-014-3564-2

**Published:** 2014-02-22

**Authors:** N. Maeda, K. Yoshimura, S. Yamamoto, A. Kuramasu, M. Inoue, N. Suzuki, Y. Watanabe, Y. Maeda, R. Kamei, R. Tsunedomi, Y. Shindo, M. Inui, K. Tamada, S. Yoshino, S. Hazama, M. Oka

**Affiliations:** 1 Department of Digestive Surgery and Surgical Oncology, Yamaguchi University School of Medicine, Ube, Yamaguchi Japan; 2 Department of Pharmacology, Yamaguchi University School of Medicine, Ube, Yamaguchi Japan; 3 Department of Immunology and Cell Signaling Analysis, Yamaguchi University School of Medicine, Ube, Yamaguchi Japan

## Abstract

**Background:**

In the tumor microenvironment, factors inhibiting the targeting of cancer cells by activated T cells have recently been noted. B7-H3 belongs to the B7 superfamily of immune regulatory ligands and plays an important role in the adaptive immune response of co-inhibitory/stimulatory factors in regulating T cells. However, the degree to which B7-H3 directly affects tumor immune evasion mechanisms remains unclear, particularly in patients with breast cancer. Regulatory T cells (Tregs) are known as a key player in the inhibition of immune mechanisms. The present study demonstrated that expression of B7-H3 on tumor cells and the number of Tregs in the tumor microenvironment independently affected prognosis in breast cancer patients.

**Methods:**

We immunohistochemically investigated the presence of B7-H3 and forkhead box P3 (Foxp3)-positive Tregs in pathological specimens from 90 patients with breast cancer.

**Results:**

Positive B7-H3 expression was associated with shorter recurrence-free survival (RFS) (*p* *=* 0.014). A higher percentage of Foxp3-positive cells also correlated with shorter RFS (*p* *=* 0.039). Multivariate analysis showed B7-H3 as an independent factor on RFS. Foxp3 expression in tumor-infiltrating lymphocytes (TILs) correlated significantly with larger tumor size (>2 cm), expression of human epidermal growth factor receptor 2 (HER2), and higher nuclear grade (*p* *=* 0.003, *p* *<* 0.001, *p* *=* 0.001, respectively). No correlation was identified between expression of B7-H3 and the percentage of Foxp3-positive TILs.

**Conclusions:**

B7-H3 and Foxp3 can be regarded as markers of poor prognosis in breast cancer. These expressions were not correlated, suggesting that B7-H3 expression plays an independent role in tumor immune evasion, regardless of Tregs.

Breast cancer is the fifth leading cause of cancer deaths among women in Japan. In attempts to control breast cancer, clarification of the tumor microenvironment will prove important. In this environment, the present study focused on interactions between tumor and immune effector cells. Several mechanisms have been suggested to result in the immune defects seen in breast cancer patients, such as a lower number of blood lymphocytes 1,2 and elevated levels of T-regulatory lymphocytes in breast cancer.^3^ B7-H3 belongs to the B7 family as a known co-inhibitory ligand.^4^ The receptor for B7-H3 has not been clearly identified, so the mechanisms underlying the effects of B7-H3 on the immune system remain unclear, particularly in terms of targeting T cells for suppression. Expression of B7-H3 protein has been detected in several tumor cell lines along with human malignancies of the lymphoma,^1^ ovary,^5^ lung,^6^ stomach,^7^ prostate,^8^ and pancreas,^9^,^10^ clear cell renal carcinoma,^11^ and colorectal carcinoma.^12^ It was reported that B7-H3 is a type1 transmembrane protein.^13^,^14^ Two possible receptors have been postulated for B7-H3 on T cells, one that gives rise to activating signals,^14^ whereas the other exerts inhibitory signals.^15^ In EL-4 lymphoma, Sun et al. 1 showed that B7-H3 displayed antitumor activity, as intratumoral injection of a B7-H3 expression plasmid led to complete regression of 50 % of lymphoma and otherwise significantly slowed tumor growth. B7-H3 mediated antitumor immunity was reported to mediate by CD8+ T cells and natural killer (NK) cells instead of CD4+ T cells. In contrast, other groups reported that treatment by intratumoral injection of an adenovirus-expressing mouse B7-H3 (Ad-B7-H3-GFP) resulted in a reduction of tumor size compared with control animals in the orthotropic murine colon cancer model.^16^ As a result, B7-H3 appears likely to exert both stimulatory and inhibitory immunological functions. Recently, B7-H3 messenger RNA (mRNA) and protein expression in breast cancer was reported.^17^ B7-H3 expression was found to correlate with the size of the primary tumor and lymphovascular invasion, as evaluated by the American Joint Committee on Cancer (AJCC) stage of breast cancer. B7-H3 also appears to be strongly expressed in breast cancer cells, with expression related to the progression of primary breast cancer to axillary lymph nodes.^17^

Regulatory T cells (Tregs) have been found to be involved in the maintenance of immune tolerance, both preventing autoimmune disease and curtailing antitumor immune response. These cells can suppress the actions of cytotoxic lymphocytes. Forkhead box P3 (Foxp3) is a member of the forkhead/winged-helix family of transcription regulators involved in regulating immune system development and function.^18^ This gene plays a crucial role in the generation of CD4+CD25+ Tregs, and the loss of Foxp3 function leads to a lack of Tregs, resulting in lethal autoaggressive lymphoproliferation, whereas overexpression of Foxp3 results in severe immunodeficiency.^19^ High levels of Tregs have been reported in the peripheral blood,^20^–^22^ lymph nodes,^23^,^24^ tumor specimens,^22^,^25^ and ascites 21 of patients with different types of cancer. The prognostic importance of Foxp3 expression in patients with breast cancer has been investigated. Foxp3 expression in breast tumor has been associated with lower probability of overall survival (OS), with increasing intensity of Foxp3 immunostaining.^26^ Foxp3 was also a strong prognostic factor for distant metastasis-free survival, but not local recurrence risk.^26^ The clinical significance of tumor infiltrating Foxp3-positive Tregs has been assessed in breast cancer patients with long-term follow-up. Bates et al. 27 indicated that high numbers of Foxp3-positive Tregs represent an important marker for the identification of breast cancer patients at risk of late relapse. They also found that the number of tumor-associated Tregs was a significant prognostic parameter for both invasive and non-invasive breast cancers that can be assessed in routinely fixed tissues by immunohistochemistry (IHC) to detect Foxp3-positive Tregs.^27^

The purpose of this study was to correlate the expression of B7-H3 and the number of Tregs in primary tumors of breast cancer. We focused on the relationship of both expressions of B7-H3 on tumor cells and infiltration of Tregs.

## Materials and Methods

### Patients and Tissue Samples

Participants comprised 90 patients with breast cancer who underwent surgery in the Department of Digestive Surgery and Surgical Oncology at Yamaguchi University Graduate School of Medicine (Yamaguchi, Japan) between April 2003 and March 2007.

Primary tumor specimens were collected from 90 patients with invasive ductal carcinoma who underwent surgery (breast-conserving surgery or total mastectomy) plus sentinel lymph node and/or axillary lymph node dissection. No patients had received any treatment before surgery. Written informed consent was obtained from all patients and the study protocol was approved by the Institutional Review Board for Human Use at Yamaguchi University School of Medicine.

Fifty-four patients (60 %) underwent breast-conserving surgery plus locoregional radiotherapy, and 36 patients (40 %) underwent modified radical mastectomy. Clinical examinations were performed every 3 or 6 months during the first 5 years according to the prognostic risk of the patient, and annually thereafter. Mammograms were performed annually. Eighty-eight patients received adjuvant therapy, consisting of chemotherapy alone in 15 cases, hormone therapy alone in 29 cases, and both in 44 cases. No human epidermal growth factor receptor 2 (HER2)-positive patients were treated with anti-HER2 therapy as an adjuvant therapy. The histological type and number of positive axillary nodes were established at the time of surgery. The malignancy of infiltrating carcinomas was scored using the histoprognostic system described by Bloom and Richardson.^28^

Estrogen receptor (ER) and progesterone receptor (PgR) statuses were determined using IHC. A tumor was considered HER2-positive from IHC with a score of 3+ or a score of 2+ with uniform intense membrane staining of >10 % of invasive tumor cells. Median follow-up was 67 months (range 7.8–90.5 months). Thirteen patients developed metastases. Recurrence-free survival (RFS) was defined as the time between initial diagnosis and first recurrence.

### Immunohistochemistry

Sections of 5 μm thickness were cut from paraffin-embedded tissue blocks, mounted on silanated slides, and subsequently dewaxed and rehydrated using xylene and graded alcohol washes. Antigen retrieval was carried out by microwaving in DAKO Target Retrieval Solution (pH 9; Dako, Glostrup, Denmark). Endogenous peroxidase was blocked for 15 min with 0.3 % hydrogen peroxide (Wako Pure Chemical Industries, Tokyo, Japan) in phosphate-buffered saline (PBS) [Oxoid, Hampshire, UK] containing 0.1 % sodium azide. After two washes in PBS, sections were blocked with serum-free protein blocker (Dako) for 10 min, followed by the addition of a primary antibody. The following primary antibodies were used in accordance with the instructions from the manufacturer: B7-H3 antibody, 1/400 dilution (MAB1027; R&D Systems, Minneapolis, MN, USA); and Foxp3 antibody, 1/100 dilution (ab20034; Abcam, Cambridge, UK). After incubation with the primary antibody, slides were washed in two changes of PBS before incubation with labeled polymer horseradish peroxidase rabbit/mouse antibody for 15 min (Envision Plus mouse/HRP system; Dako). Sections were subsequently incubated with Dako-Chromogen solution and washed in deionized water. Background staining was performed using Mayers hematoxylin and sections were then dehydrated through ascending alcohols to xylene, and mounted. To ensure antibody specificity, negative control slides were incubated with mouse immunoglobulin G1 monoclonal antibody (Abcam). Results of IHC were evaluated by scanning each slide under low magnification to identify regions containing positive immunoreactivity. Immunostaining was further evaluated at high-power magnification. Tumor samples were examined by two observers in a blinded manner.

### Scoring of B7-H3 Expression

To assess the impact of B7-H3 protein expression by cancer cells on prognosis, immunohistochemical staining was performed. Evaluation of B7-H3 staining in cancer cells was performed semiquantitatively, as described by Loos et al.^10^,^29^ This scoring method is based on the stained area and intensity of staining. Quantification was made as follows: <33 % of cancer cells—1; ≥33 to 66 % of cancer cells—2; >66 % of cancer cells—3; absent/weak staining—1; moderately intense staining—2; strong staining—3. Each section was given a final grade derived as the product of the area and intensity scores. Sections with a final score of ≤3 were classified as showing low B7-H3 expression (B7-H3 low), whereas sections with a final score >3 were classified as showing high B7-H3 expression (B7-H3 high).

### Scoring of Forkhead Box P3 (Foxp3)-Positive Cells in Tumor-Infiltrating Lymphocytes (TILs)

Absolute numbers of Foxp3-positive cells in assessable 1-μm invasive tumor cores were counted manually using an eyepiece reticule without any prior knowledge of specimen identity. The number of Foxp3-positive cells and tumor-infiltrating lymphocytes (TILs) was counted using a computerized image analysis system composed of a DP70 CCD (charge-coupled device) camera (Olympus, Tokyo, Japan) mounted on an Olympus AX70 light microscope (Olympus). Under 400× magnification, there were at least 12 independent and intact computerized microscopic fields for the duplicates of each patient sample. Eight independent microscopic fields (400×), representing the densest lymphocytic infiltrates, were selected for each patient sample to ensure representativeness and homogeneity. Numbers in the eight fields were cumulated and then averaged to calculate the final number for one computerized 400× microscopic field (0.0768 mm^2^/field). The evaluation of Foxp3-positive cells and TILs was performed by two independent observers in a blinded manner. Discrepancies in enumeration, within a range of 5 %, were re-evaluated and a consensus decision was made. The ratio of Foxp3-positive cells/TILs was calculated for each specimen. We selected the median value as the cut-off for defining TIL subgroups (median 0.097). High and low ratios of Foxp3 were termed Foxp3 high and Foxp3 low, respectively.

### Statistical Analysis

Actuarial OS and RFS rates were calculated by the Kaplan–Meier method and analyzed using the log rank test. Uni- and multivariate analyses were based on the Cox proportional hazards regression model. Secondary analysis was performed to assess the relationship between expression of B7-H3, Foxp3-positive cells and clinicopathological characteristics. For the comparison of individual variables, paired-sample *t* tests, χ^2^ tests and Mann–Whitney U tests were carried out as appropriate. Two-tailed *p* < 0.05 was judged as significant. All analyses were performed using Dr. SPSS for Windows, version 12.0 software (SPSS, Chicago, IL, USA).

## Results

### B7-H3 Expression of Breast Cancer

**Fig. 1:**
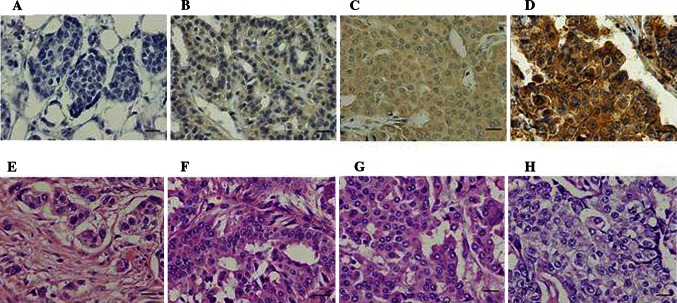
Immunohistochemical staining and scoring of B7-H3 in breast cancer tissue. B7-H3 expression is shown in both cell membrane and cytoplasm (*brown staining*). Photos demonstrate each score’s representative histopathology image. A final score of 1–3 belongs to B7-H3 low, and a final score >3 belongs to B7-H3 high. **a** No B7-H3 expression in breast cancer (a final score of 1; B7-H3 low). **b** Week intensity of staining in >66 % of breast cancer (a final score of 3; B7-H3 low). **c** Moderate intensity of staining in >66 % of breast cancer (a final score of 6; B7-H3 high). **d** High intensity of staining in >66 % of breast cancer (a final score of 9; B7-H3 high). **e–h** Hematoxylin/eosin staining is shown in **a–d**. Magnification for all, ×40 (scale bar 20 μm)

B7-H3 protein expression was found in the cytoplasm of breast tumor cells. Ninety tissue sections from patients with breast cancer were examined. B7-H3 expression on primary carcinoma cells was detected at various levels, and was not detected in seven patients (8 %; Fig. 1a). Weak expression was seen in 26 patients (29 %; Fig. 1b), moderate expression in 29 patients (32 %; Fig. 1c), and strong expression in 28 patients (31 %; Fig. 1d). Depending on the area of positive immunoactivity, a final overall score (high or low B7-H3) was established as described in the “Material and Methods” section. A total of 58 % of tumor samples were identified as B7-H3 high, while 42 % showed B7-H3 low. No significant associations were identified between B7-H3 expression and pathological factors (Table 1).

**Table 1:** Correlation of B7-H3 expression in breast cancer cells and Foxp3-positive cells in TILs with clinicopathologic features in 90 patients

Clinical parameters	No. of cases	Foxp3-positive cell/TIL			B7-H3 expression		
		Low (%)	High (%)	*p* value^a^	Low (%)	High (%)	*p* value^a^
Age (years)							
≤50	38	21 (23.3)	17 (18.9)	0.673	12 (13.3)	26 (28.9)	0.641
>50	52	26 (28.9)	26 (28.9)		26 (28.9)	26 (28.9)	
Tumor size (cm)							
≤2	40	28 (31.1)	12 (13.3)	**0.003**	17 (18.9)	23 (25.6)	1.000
>2	50	19 (21.1)	31 (34.4)		21 (23.3)	29 (32.2)	
Nodal metastasis							
Without	49	37 (41.1)	21 (13.3)	**<0.001**	23 (25.6)	26 (28.9)	0.393
With	41	10 (11.1)	31 (34.4)		15 (16.7)	26 (28.9)	
Hormone receptor							
Positive	73	41 (45.6)	32 (35.6)	0.178	28 (31.1)	45 (50.0)	0.173
Negative	17	6 (6.7)	11 (12.2)		10 (11.1)	7 (7.8)	
HER2							
Positive	40	13 (14.4)	27 (30.0)	**0.001**	18 (20.0)	22 (24.4)	0.672
Negative	50	34 (37.8)	16 (17.8)		20 (22.2)	30 (33.3)	
Nuclear grade							
Low (0,1)	34	25 (27.8)	9 (10.0)	**0.002**	17 (18.9)	17 (18.9)	0.277
High (2,3)	56	22 (24.4)	34 (37.8)		21 (23.3)	35 (38.9)	
Vascular invasion							
Positive	58	32 (35.6)	26 (28.9)	0.451	24 (26.7)	34 (37.8)	0.828
Negative	32	15 (16.7)	17 (18.9)		14 (15.6)	18 (20.0)	

*p* values less than 0.05 are in bold

*Foxp3* forkhead box P3, *TILs* tumor-infiltrating lymphocytes, *HER2* human epidermal growth factor receptor 2

^a^Chi-square analysis

### Foxp3-Positive Cells in TILs of Breast Cancer

**Fig. 2:**
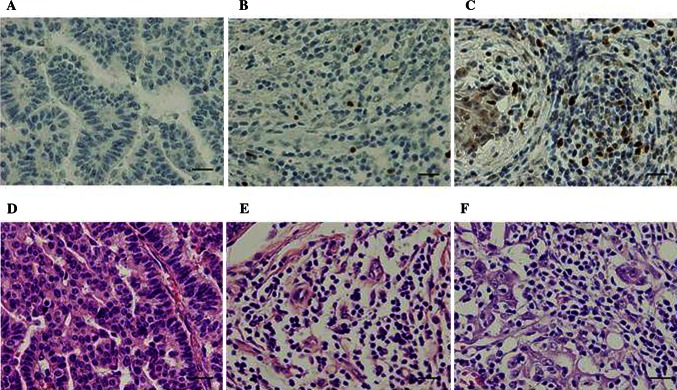
Immunohistochemical detection of Foxp3-positive cell in breast carcinoma tissue. Photos demonstrate each group of representative histopathology image. The ratio of Foxp3-positive cells/TILs was categorized by median value (median 0.097) into high and low ratios of Foxp3 (Foxp3 high and low). **a** Control. **b** Foxp3 low in tumor infiltrating lymphocytes (Foxp3-positive cell/TIL; 0.032). **c** Foxp3 high in tumor infiltrating lymphocytes (Foxp3-positive cell/TIL; 0.11). **d–f** Hematoxylin/eosin staining is shown in **a–c.** Magnification for all, ×40 (scale bar 20 μm). *Foxp3* forkhead box P3, *TILs* tumor-infiltrating lymphocytes

Lymphocytes infiltrating within tumors presented in a diffuse pattern and those in tissue surrounding tumors were more abundant and tended to form lymphoid aggregates (Fig. 2). Foxp3 high was observed significantly more often in tumors with positive nodal status (*p* *<* 0.001), large tumor size (*p* *<* 0.05), high histological grade (*p* *=* 0.002), or HER2 overexpression (*p* *=* 0.001). No significant associations were identified between Foxp3-positive cell infiltration and either ER/PgR expression or lymphovascular invasion (Table 1).

### B7-H3 Expression Correlates with Prognosis

**Fig. 3:**
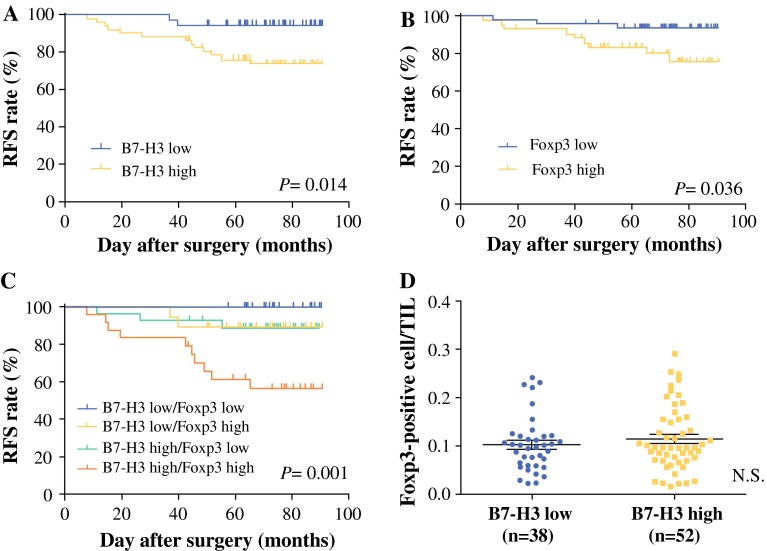
Correlation of RFS with B7-H3 expression in breast cancer cells and Foxp3-positive cells in TILs. **a** B7-H3 high (*n* = 52) was associated with significantly reduced RFS in patients with breast cancer (*p* *=* 0.0137; HR 0.2781; 95 % CI 0.1005–0.7696). **b** Patients with Foxp3 high (*n* = 43) had significantly improved RFS compared with Foxp3 low (*p* *=* 0.0368; HR 0.2974; 95 % CI 0.0953–0.929). **c** Further categorized patients into four groups: B7-H3 high/Foxp3 high; B7-H3 high/Foxp3 low; B7-H3 low/Foxp3 high; and B7-H3 low/Foxp3 low. Patients with B7-H3 high/Foxp3 high relapsed within a shorter period than patients with B7-H3 low/Foxp3 low (*p* *=* 0.0014; HR 0.1325; 95 % CI 0.0382–0.4596). **d** The ratio of Foxp3-positive cells in TILs and B7-H3 protein expression in breast cancer cells. No significant difference was found in the ratio of tumor-infiltrating Foxp3-positive cells between B7-H3 high and B7-H3 low (*p* *=* 0.532). This correlation was tested by the Mann–Whitney U test. *RFS* recurrence-free survival, *Foxp3* forkhead box P3, *TILs* tumor-infiltrating lymphocytes, *HR* hazard ratio, *CI* confidence interval

B7-H3 high was associated with significantly reduced RFS in patients with breast cancer [*p* *=* 0.0137; hazard ratio (HR) 0.2781; 95 % confidence interval (CI) 0.1005–0.7696; Fig.3a). Five-year RFS rate of patients with B7-H3 low was 94.7 % in contrast to 76.3 % in patients with B7-H3 high. However, OS was not associated with expression of B7-H3 (*p* *=* 0.5660; HR 3.119; 95 % CI 0.5325–18.27; data not shown). Five-year OS rate was 100 and 97.7 % in B7-H3 low and high patients, respectively (median survival time of B7-H3 high: 89.5 months).

### Ratio of Foxp3-Positive Cells in TILs of Breast Cancer Correlates with Prognosis

Foxp3-positive cell/TIL ratio was associated with RFS in breast cancer (*p* *=* 0.0368; HR 0.2974; 95 % CI 0.09525–0.9286; Fig. 3b). However, Foxp3-positive cell/TIL ratio did not correlate with OS (*p* *=* 0.599; HR 0.5412; 95 % CI 0.055–5.323; data not shown).

### Combined Prognosis with Expression of B7-H3 and Foxp3-Positive Cells in TILs

As previously described, B7-H3 expression score did not correlate with the percentage of Foxp3-positive cells among TILs, but each value was associated with RFS. We therefore further categorized patients into four groups: B7-H3 high/Foxp3 high (*n* = 24); B7-H3 high/Foxp3 low (*n* = 28); B7-H3 low/Foxp3 high (*n* = 19); and B7-H3 low/Foxp3 low (*n* = 19) (Fig. 3c). Patients with B7-H3 high/Foxp3 high relapsed within a shorter period than patients with B7-H3 low/Foxp3 low (*p* *=* 0.001; HR 0.1325; 95 % CI 0.0382–0.4596; Fig. 3c). Interestingly, no B7-H3 low/Foxp3 low patients showed recurrence. In the group of B7-H3 low, no significant difference in RFS was seen between Foxp3 high and low subgroups.

### Multivariate Analysis

Multivariate analysis showing HR for patient RFS conferred by nodal status, tumor size, nuclear grade, higher numbers of Foxp3-positive TIL and higher expression of B7-H3. Expression of B7-H3 were revealed as independent prognostic factors for RFS (*p* *=* 0.025; HR 8.5; 95 % CI 1.233–24.269; Table 2).

**Table 2:** Multivariate analyses showing hazard ratio for patient RFS conferred by tumor size, nodal status, nuclear grade, vascular invasion, hormone receptor, HER2, B7-H3 expression, Foxp3-positive cell in TILs

Variable	Hazard ratio	95 % CI	*p* value^a^
B7-H3 expression (high vs. low)	5.471	1.233–24.269	0.025
Foxp3-positive cell in TILs (high vs. low)	3.416	0.929–12.564	0.065
Lymphovascular invasion (+ve vs. ^–^ve)	5.405	0.670–43.588	0.113
Tumor size (≤2 vs. >2 cm)	1.798	0.449–7.199	0.407
Nodal status (+ve vs. ^–^ve)	1.588	0.468–5.390	0.458
Nuclear grade (grade 1 vs. grade 2,3)	0.669	0.105–4.259	0.671
Hormone receptor (+ve vs. ^–^ve)	0.779	0.192–3.161	0.726
HER2 (+ve vs. ^–^ve)	1.070	0.351–3.260	0.905

*RFS* recurrence-free survival, *HER2* human epidermal growth factor receptor 2, *Foxp3* forkhead box P3, *TILs* tumor-infiltrating lymphocytes, *+ve* positive, ^–^ *ve* negative, *CI* confidence interval

^a^Cox’s proportional hazards regression analysis

Correlation between B7-H3 Expression and Tumor-Infiltrating Foxp3-Positive Cells

No significant difference was found in the percentage of tumor-infiltrating Foxp3-positive cells between B7-H3 high and B7-H3 low (*p* *=* 0.532, Mann–Whitney U test; Fig.3d).

## Discussion

This study demonstrated that the numbers of Foxp3-positive cells was significantly increased among tumors with positive nodal status, large tumor size, high histological grade, and HER2 overexpression. Although B7-H3 has been associated with downregulation and evasion of host immunity, the clinical and functional significance of this protein remains unclear. Our study suggested that B7-H3 expression in breast cancer was unrelated to clinical and pathological parameters. Patients with B7-H3 high/Foxp3 high relapsed within a shorter period than patients with B7-H3 low/Foxp3 low. No significant difference in terms of RFS was evident between B7-H3 high/Foxp3 high and B7-H3low/Foxp3 high.

Recent studies of B7 family proteins named B7-H1 (PD-L1), B7-DC (PD-L2), inducible costimulatory molecule (ICOSL) and B7-H3 have focused on both tumor immunity effects and immune evasion. B7-1 and B7-2 are the representative proteins of the B7 family and are expressed in antigen-presenting cells (APCs). However, B7-H1 and B7-H3 are expressed in multiple organs as well as in APCs, indicating that these molecules potentially act as immune-modulators at the sites of inflammation.^13^,^30^ Human B7-H3 is induced in dendritic cells and monocytes by inflammatory cytokines. B7-H3 binds to an unknown receptor expressed on activated CD4+ and CD8+ T cells.^14^ This receptor is distinct from CD28, CTLA-4, PD-1 and ICOS34, the receptors known to bind to other B7 family proteins. B7-H3 has been reported as a negative regulator that preferentially downregulates T helper type 1-mediated immune responses.^13^ On the other hand, another report suggests an opposite function, with human B7-H3 augmenting TCR-mediated T-cell proliferation, interferon-γ production and generation of cytotoxic T lymphocytes (CTLs) in vitro, indicating that B7-H3 may have positive regulatory functions in CTL responses.^14^

B7-H3 expression has been found in a variety of human malignancies, including breast, prostate, non-small cell lung, pancreatic, gastric, endometrial, and colorectal cancers.^6^–^8^,^10^,^12^,^17^,^31^–^34^ It was reported that B7-H3 expression by breast cancer cells are a potential tumor progression factor that is a predictor of early regional nodal metastasis.^17^

In non-small cell lung cancer, Sun et al. 6 suggested B7-H3 as a factor related to lymph node metastasis. In prostate cancer, patients with high levels of B7-H3 expression displayed a worse prognosis than those with low levels of B7-H3 expression.^8^ We suspect that the coregulatory molecule B7-H3 might play a very similar role in breast cancer. In this study, high expression of B7-H3 was significantly associated with shortened RFS in breast cancer. However, B7-H3 expression did not correlate with any other clinicopathological factors, such as tumor size, axillary nodal status, ER expression, or HER2 overexpression. Further identification and understanding of the B7-H3 signaling pathway and potential receptors may offer new therapeutic strategies for primary breast cancer.

Tregs are present in the tumor microenvironment, inhibiting autologous T-cell proliferation. Tregs are associated with suppression of antitumor immunity. Increasing numbers of Tregs have been reported in several human cancers, including not only breast cancer 27 but also lung,^35^ pancreas,^20^,^36^,^37^ and ovarian tumors.^38^ Tumor infiltration by Tregs has been shown to correlate significantly with worse prognosis among breast cancer patients.^27^ Foxp3 is a member of the forkhead box family of transcription factors and was initially thought to be a master regulatory gene for lineage commitment or development of CD4+CD25+ Tregs.^19^ Foxp3 remains the best single marker of Tregs.^39^

Immunosuppressive Foxp3-positive Tregs have been linked to poor response to chemotherapy and poor prognosis among breast cancer patients. Decreased peritumoral Tregs offer an independent predictor of pathological complete response (pCR), while intratumoral Tregs after chemotherapy have been associated with both OS and progression-free survival. Peritumoral Tregs are sensitive to chemotherapy and associated with pCR, while intratumoral Tregs offer an independent prognostic predictor for breast cancer patients.^40^

In our results, B7-H3 and Treg expressions were associated with poor prognosis among breast cancer patients. Although no correlations with these expressions were seen, patients with both B7-H3 high and Treg high showed the worst prognosis, whereas patients with B7-H3 low and Treg low showed no recurrence at all. These data suggest that examination of both B7-H3 and Treg expression may offer a more accurate prediction of prognosis than the expression of either molecule alone. We recognized that these biomarkers are potentially able to be a new therapeutic target.

The blockade of B7-H1 known as PD-L1 is under clinical trials all over the world at the present time. Using this approach, because B7-H3 has a high homology of B7-H1, blockade of these molecules is supposed to be of high feasibility. In addition, downregulation of Treg can be achievable by low-dose cyclophosphamide.^41^ Previous studies have demonstrated that patients with ER/PgR-positive cancer frequently relapsed with non-visceral disease. Most patients could, for a time, be controlled for treatment by endocrine therapy. In this study, 73 patients (81 %) had ER positive tumors. Therefore, there were no significant differentiations between these expressions and OS. Many analyses showed that breast cancer subtypes were associated with the prognosis, and ER-positive patients relapsed 5 years after initial therapy.^42^,^43^ In this study, the median follow-up duration was 67 months. Although this period is no longer of great significance, we consider that it is not too short in assessing the prognosis.

## Conclusions

This study showed that detection of B7-H3 on breast cancer and Tregs among infiltrating T cells predicts worsened prognosis. Even in slow-growth-type cancers such as breast cancer, tumor immune evasion through B7-H3 expression on tumor cells and/or Treg infiltration acts as an important factor in determining prognosis.
